# Health data hubs: an analysis of existing data governance features for research

**DOI:** 10.1186/s12961-023-01026-1

**Published:** 2023-07-10

**Authors:** Celia Alvarez-Romero, Alicia Martínez-García, Máximo Bernabeu-Wittel, Carlos Luis Parra-Calderón

**Affiliations:** 1 Computational Health Informatics Group, Institute of Biomedicine of Seville, IBiS/Virgen del Rocío University Hospital/CSIC/University of Seville, Avenue Manuel Siurot S/N, 41013 Seville, Spain; 2 Internal Medicine Department, Virgen del Rocío University Hospital, Seville, Spain

**Keywords:** Health data management, Health data infrastructure, Health data hub, Patterns of governance, Governance models, Survey

## Abstract

**Background:**

Digital transformation in healthcare and the growth of health data generation and collection are important challenges for the secondary use of healthcare records in the health research field. Likewise, due to the ethical and legal constraints for using sensitive data, understanding how health data are managed by dedicated infrastructures called data hubs is essential to facilitating data sharing and reuse.

**Methods:**

To capture the different data governance behind health data hubs across Europe, a survey focused on analysing the feasibility of linking individual-level data between data collections and the generation of health data governance patterns was carried out. The target audience of this study was national, European, and global data hubs. In total, the designed survey was sent to a representative list of 99 health data hubs in January 2022.

**Results:**

In total, 41 survey responses received until June 2022 were analysed. Stratification methods were performed to cover the different levels of granularity identified in some data hubs’ characteristics. Firstly, a general pattern of data governance for data hubs was defined. Afterward, specific profiles were defined, generating specific data governance patterns through the stratifications in terms of the kind of organization (centralized versus decentralized) and role (data controller or data processor) of the health data hub respondents.

**Conclusions:**

The analysis of the responses from health data hub respondents across Europe provided a list of the most frequent aspects, which concluded with a set of specific best practices on data management and governance, taking into account the constraints of sensitive data. In summary, a data hub should work in a centralized way, providing a Data Processing Agreement and a formal procedure to identify data providers, as well as data quality control, data integrity and anonymization methods.

**Supplementary Information:**

The online version contains supplementary material available at 10.1186/s12961-023-01026-1.

## Background

The study presented in this manuscript was carried out during the Coordination and Support Action HealthyCloud (Health Research & Innovation Cloud), which has received funding from the European Commission. It started in March 2021 and will finish in August 2023.

The main aim of HealthyCloud [1] is to align all the knowledge and expertise in health data spread across European and international actors, as well as to lay the foundations for the future European Health Research and Innovation Cloud (HRIC) [2]. HRIC will become a fundamental part of the European Health Data Space (EHDS) [3]. The HRIC will enable the secondary use of data and the capabilities to analyse and share data to drive the limits of health research within an ethically and legally compliant framework that builds and reinforces the trust of patients and citizens.

The digitalization of health systems represents an essential opportunity for health research activities. An enormous amount of health-related data are now generated and collected within healthcare systems [4, 5]. Research networks have assembled and curated health data, at the patient and/or population levels, for multiple diseases in the form of cohorts. In addition, dedicated research infrastructures in Europe have long harmonized the collection and preservation of specific biological specimens and promoted the development of clinical trials, aiming to reuse the results by other researchers [6]. The reuse of health data is a fast-growing field recognized as essential to realizing the potential for high-quality healthcare, improved healthcare management, reduced healthcare costs, population health management and effective health research [7]. However, the dispersed nature of data generation and the challenge of addressing ethical and legal concerns relating to the collection and use of sensitive data represent significant barriers to the use of health data [8, 9]. The significant technical barriers in terms of the need for more structured information and limited interoperability between different health fields also affect the full exploitation of health data for research purposes. Stronger and better-aligned approaches to health data governance are required to facilitate data sharing and reuse whilst addressing ethical and legal barriers [10, 11]. In addition, a recent study has concluded that funding agencies do not support data sharing mandates because of data protection regulations. That is, the need for global standards and guidelines for health data is recognized [12]. However, policy measures that restrict the authority of researchers to make data sharing decisions are often not supported. In this regard, incentive design is paramount if funding agencies do not wish to impose restrictions on the decision-making authority of researchers [13]. Additionally, the call for stronger health data governance is gaining significant movement with the progress of digital transformation [14–16].

In this sense, HealthyCloud execution has included capturing different governance and auditing models behind data hubs across Europe and managing health data to analyse the existing initiatives related to domain-specific data hubs. For this purpose, the definition of important terms related to this study was discussed.

Firstly, in the HealthyCloud project, a health data hub is defined as a data infrastructure with the following minimal inclusion criteria [17]: (i) a digital technical infrastructure with the core mission of enabling health data sharing; (ii) providing health data from a different source; (iii) allowing for the discovery of health datasets; (iv) having a metadata discovery service; (v) having a data accessibility mechanism following existing regulation; and (vi) having an authorization functionality, provided by the same data hub or by an external institution.

Secondly, HealthyCloud defines data governance as the ‘assembly of policies and processes, coordination aspects, data usage and accessibility principles and data management procedures for a certain health data infrastructure to ensure legal compliance, consistency and good data quality throughout the different stages of the data life cycle’ [17].

## Methods

The study described in this manuscript covers an analysis of health data governance patterns generated after identifying commonalities in the governance models of existing data hubs. To understand how health data are managed by dedicated infrastructures called data hubs [18, 19], a capture of the different data governance behind health data hubs across Europe was carried out. Existing initiatives and projects related to domain-specific data hubs at regional, national, European and global levels were analysed and identified on the basis of previous experiences and contacts, as well as literature and internet searchers. Afterwards, a list of 99 representative data hubs in Europe was collected.

To gather the feedback from the representative data hubs collected, a collaborative survey was designed, including the contributions and improvements detected by the HealthyCloud researchers. The survey’s main objectives were (i) to evaluate the feasibility of linking individual-level data between data collections and (ii) to perform a landscape analysis of the different governance models in those data infrastructures. The survey was developed in an electronic tool (Typeform.com).

The survey included questions related to administrative information such as name of the data hub, data controller, website, and features, data storage and capacity, type of source and data, level of data aggregation, use of anonymization methods, completeness of data infrastructure (e.g. geographical coverage), data quality aspects (e.g. use of data quality control or error checking), legal aspects, sustainability and governance. Additional file 1 includes the whole questionnaire that was sent to respondents.

**Fig. 1:**
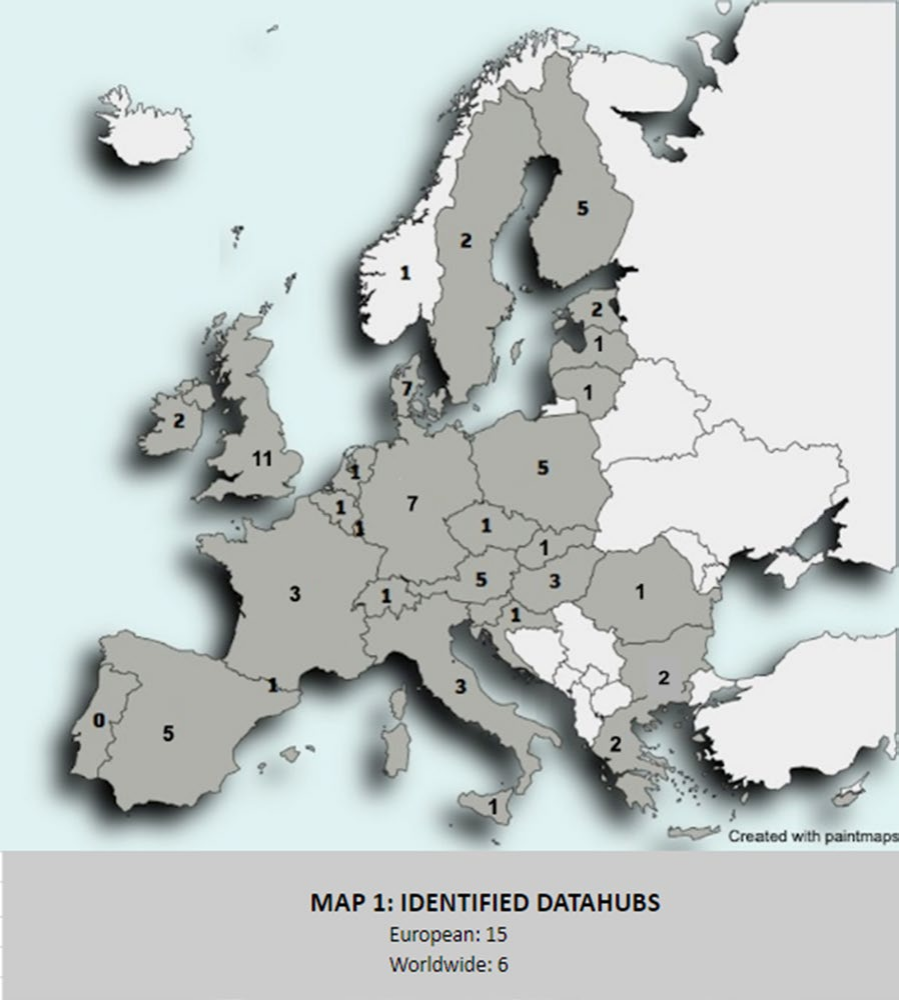
Map showing the number of data hubs contacted in each country shaded. In addition, the bottom of the map indicates the number of data hubs identified with a European or global geographical coverage (not just nationally as indicated in the countries on the map)

Finally, the survey was sent at the beginning of January 2022 to the target audience, which was the national, European, and global data hubs identified previously. Concretely, the survey was sent to a representative list of 99 data hubs (see Fig. 1) after the effort to ensure a robust representation of all the data hubs in Europe.

**Fig. 2:**
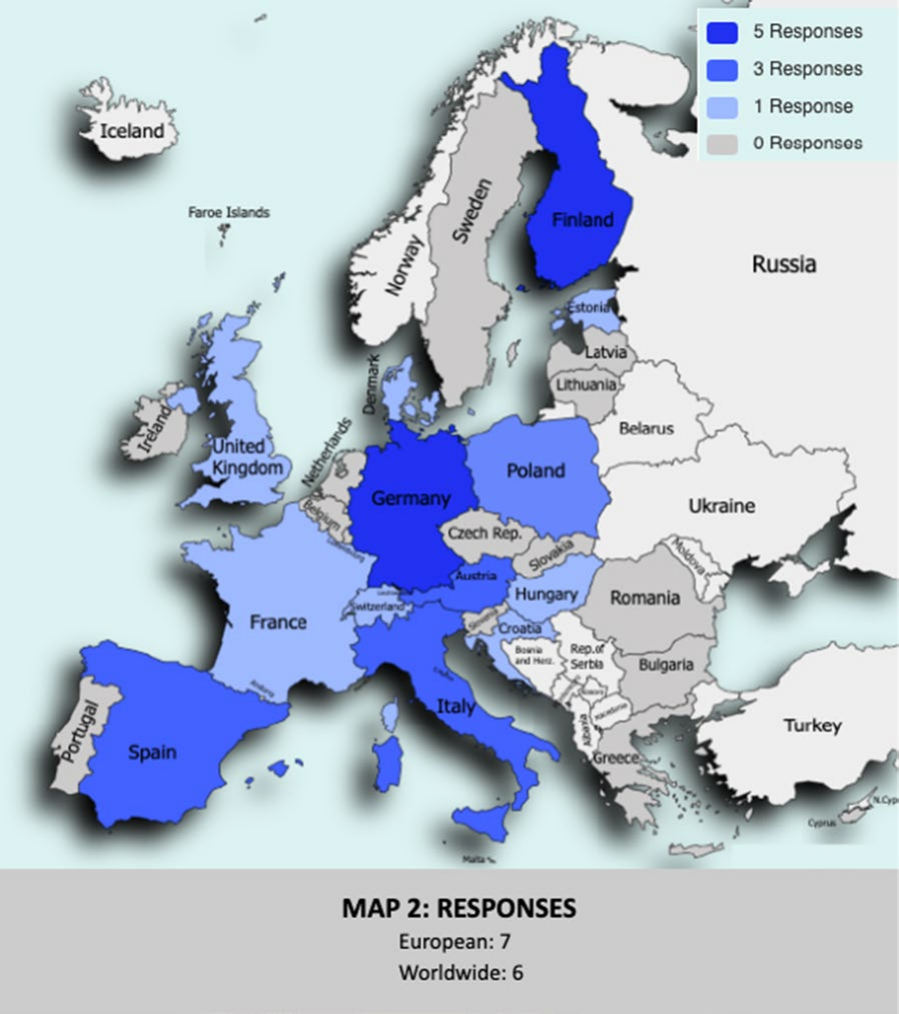
Map showing the countries from which these data hubs finally responded to the survey. In addition, at the bottom of the map the number of data centres that responded and whether their geographical coverage was European or global is shown

Finally, 41 out of the 99 (41%) contacted data hubs answered the survey until the cutoff date of June 2022. Additionally, a detailed list of the 41 respondents’ data hubs is detailed as part of the Additional file 1. Figure 2 shows the final geographical coverage achieved through the survey responses.

All the material collected through the survey was analysed (both structured and free-text questions), focusing on identifying actors, data aspects and business processes involved in the hubs’ governance, also considering ethical, legal, societal impact (ELSI) aspects.

To appropriately cover the different levels of granularity identified in some data hubs’ characteristics (such as kind of data hub organization, role, etc.), stratifications (i.e. segmentation of the responses to be analysed) were performed using characteristics such as the kind of data hub organization (centralized versus federated), and the role applied in data management (data controller versus data processor), delivering specific profiles.

Finally, a set of best practices related to data governance patterns were identified by analysing the list of the most frequent aspects of data hub respondents. In fact, to strengthen the results of this study, the list of best practices was validated with the data hubs interviewed, involving them in the review phase of the best practices generated.

## Survey overview

Through the analysis of the survey responses, the results of this study expect to capture the different data governance behind health data hubs across Europe. Concretely, the survey conducted was focused on analysing the feasibility of linking individual-level data between data collections and the generation of health data governance patterns.

A survey results overview is shown below to contextualize the results of the study which will be presented later in the manuscript.

During the analysis, to improve readability, the decimal places were considered not representative, so all percentages were rounded without using decimal places, taking into account that with 41 responses, the minor step (1 answer more or less) is more than 2%.

### Data hub criteria

Apart from the characteristics defined for the health data hub concept [17], from the multiple-choice question survey responses, 27 respondents added to this minimal inclusion criteria the feature ‘a digital platform that receives and stores data’, 30 added the feature ‘it receives data from a single source and/or multiple sources’, and 26 added the feature ‘it has control over the data stored’.

### Data hub main features

All data hubs provided their official titles and websites. On several of the websites, a Data Governance section is included in the website. This finding is an important best practice included in the patterns of governance.

Regarding the data infrastructure organization, 22% answered ‘it has a decentralized management’ and 70% answered ‘it is managed centrally’; the rest did not apply or did not answer.

### Data management

Concerning anonymization, 65% (26) of the respondents stated anonymization methods are used in those data hubs: 8 data hubs answered that they use anonymization methods at the point of collection, 3 before sharing them internally, 11 before sharing them externally, 3 at the point of publishing, and 1 not specified. Furthermore, 20% (8) do not anonymize data in those data infrastructures. This question does not apply to 15% (6) of the respondents.

Regarding whether anonymization is performed by the data infrastructure and/or the data are received already anonymized, this question was not answered by the 35% that in the previous item stated they do not anonymize data or the question does not apply to them. Of the 25 responses with information, 48% of these data hubs perform the anonymization and 24% receive anonymized data. Both events occur in 28% of the 25 data hubs. Concerning pseudonymization, it has to be noted that 80% of the respondents have pseudonymized data, versus 7% who do not, while 10% added that the question does not apply to their data infrastructure, and 2% stated that they did not know.

### Data quality aspects

A total of 83% of the respondents stated that data quality controls are applied in their data hubs, 7% that they do not use data quality controls and 10% that they did not know. Another notable finding is that only 17 out of 38 respondents stated data are only included if it reaches a certain quality level, with 6 out of 38 respondents stating that they do quality control for internal use only, and 7 out of 38 that minimum levels of quality of the data are not needed for the data to be included in the data infrastructure, but the results of the quality control are available when searching for the data. Finally, 6 out of 38 chose the option does not apply and 2 out of 38 answered ‘unknown’.

Another aspect related to data quality is checking for errors and completeness: 61% of the respondents stated that a tool is used for error checking, compared with 24% who do not, and 2 out of 41 respondents (5%) answered that they do not know and 4 out of 41 (10%) stated that the question does not apply to that data infrastructure. Out of the 25 who previously stated that a tool is used for error checking, 21 (84%) specified the tool they use, and 7 out of 25 (28%) specified the checksum technique in their answer.

Furthermore, keeping track of the versions is very common for the data hubs that answered the survey: 24 out of 41 (59%) stated that they have a process to keep track of the different versions of the datasets, versus 8 out of 41 (20%) that stated they do not have this kind of process. In addition, 8 out of 41 (20%) answered that the question does not apply to that data infrastructure and 1 out of 41 (2%) answered that they did not know. Out of the 24 who stated that they keep track of the version process, 19 (79%) specified the process they use.

### Data management

The survey asked if there was a formal procedure to know who provides the data. While 4 of the 41 respondents did not complete this question, the remaining 37 answers included 16% stating they do not use a formal procedure to know who provides the data, and 84% stating they do. For these, the survey asked about specific procedures (i.e. contracts, agreements, open information in the organization), obtaining in the responses several specific procedures: legal contracts, different kinds of agreements (collaboration, accreditation data access, confidentiality, data transfer, data sharing, data processing, use, deposition, etc.), regulations, open information in the organization, queryable resource information on data access and data reuse conditions, terms of use, licences, user needs to register, mandatory institute email address, information about the principal investigators and the project, alliance membership, assigned Data Access Committee and data permissions based on the act on a secondary use.

Related to a Data Access Agreement (DAA) to be signed between data providers and data requesters, 38 of 41 respondents completed this question, with 55% (21) of the 38 interviewed data hubs’ providing a DAA, 24% not, and 21% selecting ‘other’, stating, amongst others, that it depends on the specific resource queried or that only employees access the data directly. In total, 52% of the 21 with DAA use a non-negotiable DAA form, and 48% provide a DAA template that may be modified under the agreement. In terms of a Data Processing Agreement (DPA) to be signed with the data providers, 38 of 41 respondents completed this question: 47% (18) of these provide a DPA, 32% do not, and 21% detailed other options, such as having pending to cover the DPA management. A total of 39% of the 18 with DPA use a non-negotiable DPA form, and 61% provide a DPA template which may be modified under the agreement. In regard to a Data Protection Impact Assessment (DPIA) model, 36 of 41 respondents answered, with 56% of the 36 data hubs using a DPIA model, and 44% not using one.

### Funding

As part of the sustainability plan, the survey included items regarding the type of funding and the sustainability plan of this current funding. In total, 38 of 41 answers respondents completed this question: national funding for the hub core function (66%), participation in projects (16%), European or international funding (11%) and private funding (8%). Concerning the sustainability plan, 42% received stable funding (of which 39% stated this stable funding is of national origin), 13% presented funding from private profits (i.e. data licence fees, pay for customer use, etc.), 32% were applying to infrastructure funding (national, European, and/or international) and 6% stated their plan to apply for competitive plans or projects related to research funding. Related to the geographical scope of this funding, including stable, non-stable, and expected profits, 77% of the data hubs stated they received funding from regional or national organizations and 32% from European or international organizations (42% of the data hubs did not specify the geographical scope, so these numbers could be biassed).

### Other data governance aspects

Concerning a catalogue of the different data sources, 34 of 41 data hubs respondents completed this question: 21% did not offer this kind of catalogue, because this specific data hub was connected only to a unique data source, and 79% provided a catalogue of different data sources.

In terms of the process to connect with the external data, a specific data hub could receive and store the data (centralized), or could link to the data remaining in the original place (federated). In total, 39 of 41 data hubs completed this question: 77% and 23% stated they are a centralized or federated data hub, respectively.

Related to the standard operating procedures (SOPs) that the data hub’s organizations follow and update regularly, 34 of 41 respondents answered this question, stating that 79% use and 21% do not use these kinds of procedures.

## Stratification depending on the kind of data hub organization

To cover this stratification, the question ‘How is the data infrastructure organized?’ was analysed. From the 41 surveyed data hubs, 40 answered and 1 did not answer. Of these 40 responses, 30 (75%) answered ‘it is managed centrally’, 9 (22%) answered ‘it has a decentralized management’, and 1 (2%) ‘this does not apply to this data infrastructure’.

On ‘data hubs managed centrally’, 23 had control of the data stored. In addition, 25 received and stored data from a single source and/or from multiple sources; 90% pseudonymized data, 90% applied data quality control, 81% established standard operating procedures (SOPs) that the organization followed and updated regularly, 89% had a formal procedure to know who provided the data, and 83% required legal approval for the data.

On ‘data hubs with decentralized management’, they may not have a single data controller and may not have a data management strategy; 9 (all of them) allowed for the discovery (findability) of health datasets and 8 were a digital technical infrastructure with the core mission of enabling health data sharing. In addition, 8 host data came from ‘patient groups’, 7 from ‘general population’ and 7 from ‘experimental settings’. In 7, the data was stored in XML format.

## Stratification depending on the role

For this stratification, the question ‘What is your organization’s role in relation to personal data?’ was analysed. From the 41 surveyed data hubs, 39 answered and 2 did not answer. Of these 39 answers, 11 (28%) answered ‘data controller’, 11 (28%) answered ‘data processor’, 12 (33%) answered ‘we have different roles in different situations’ and 4 (10%) answered ‘none of the above’.

Regarding ‘data controller’, 82% were managed centrally, 100% pseudonymized data, 10 had control over the data stored, and 9 received data from a single source and/or multiple sources. Additionally, 9 of them had data from the ‘general population’, 82% had a process to keep track of the different versions of the datasets and 90% had a formal procedure to know who provided the data; 81% established SOPs that the organization followed and updated regularly and 81% provided a catalogue of the different data sources.

Regarding ‘data processor’, 80% were managed centrally, 80% had pseudonymized data, and 9 were a digital platform that received and stored the data. Furthermore, 90% had an authorization functionality provided by the organization itself or by an external institution, and 90% had a data accessibility mechanism in accordance with existing regulations. In addition, 91% had a formal procedure to know who provided the data, and 80% had established SOPs that the organization followed and updated regularly.

## Results

A general pattern of data governance for data hubs is defined below using the conclusions obtained in the analysis of the 41 survey responses. To define the general pattern of governance, a common characteristic was considered if the respondents coincided by at least 60%.

Hereafter, specific profiles are defined, generating data governance patterns for data hubs, using the conclusions obtained in the stratifications in terms of the kind of organization (centralized versus decentralized) and role (controller or processor). Specific data governance patterns are counted from 75% (prevalence). This percentage needed to be reduced in the case of the general one (from 75% to 60%), because when all the responses were analysed together, fewer commonalities were found. For each pattern of data governance (both the general one and the specifics), data aspects, business models, and ELSI aspects were defined, preceded by the list of actors involved in these processes.

### Most frequent aspects

After performing the analysis of the 41 responses of the survey, the most frequent aspects are listed below.

**Fig. 3:**
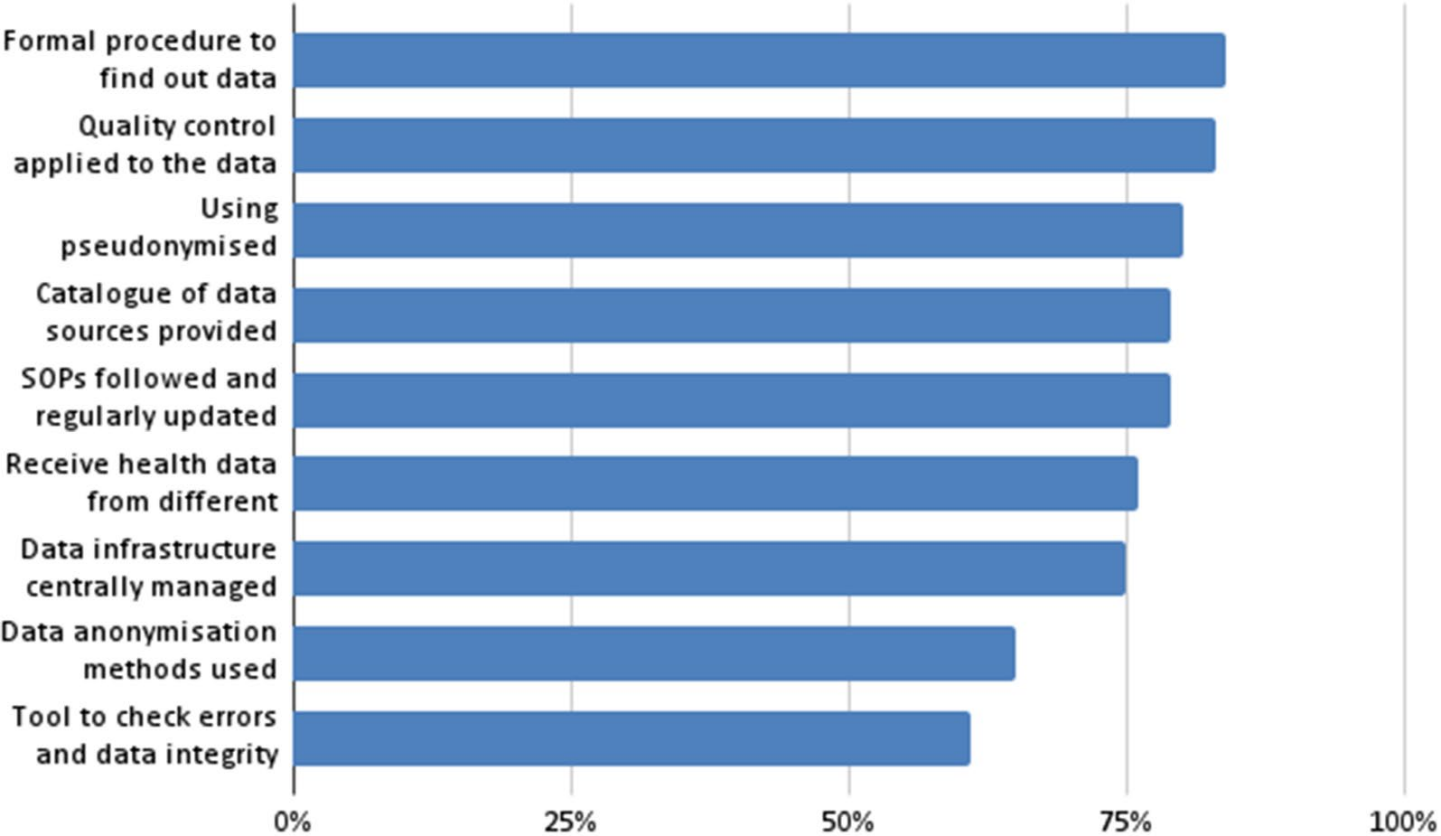
Most frequent aspects (simple-choice questions with percentages)

Concerning the simple-choice questions (with percentages): (i) formal procedure to find out who provides the data (84%); (ii) quality control is applied to the data (83%); (iii) a catalogue of the different data sources is provided (79%); (iv) there are standard operating procedures (SOPs) that are followed and regularly updated (79%); (v) they receive health data from different sources (76%); (vi) the data infrastructure is centrally managed (75%); (vii) data anonymization methods are utilized (65%), using pseudonymized data (80%); and (viii) a tool is used to check for errors and data integrity (61%). Figure 3 shows a graphical representation of these percentages.

**Fig. 4:**
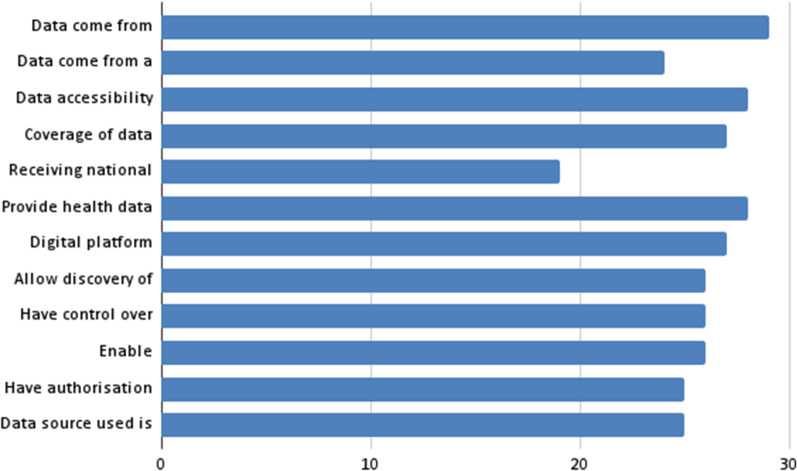
Most frequent aspects (multiple-choice questions with absolute values)

Regarding multiple-choice questions (with absolute values): (i) data come from the general population (29) or from a group of patients (24); (ii) a data accessibility mechanism is available in accordance with current regulations (28); (iii) the coverage of the data infrastructure is national (27), receiving national funding (19); (iv) they provide health data from different sources (28); (v) they are a digital platform that receives and stores data (27); (vi) they allow for the discovery (findability) of health data sets (26); (vii) they have control over stored data (26); (viii) they enable discoverability of health datasets (26); (ix) they have authorization functionality, provided by the organization itself or by an external institution (25); and (x) the type of data source used is the electronic health record (EHR) (25). Figure 4 shows a graphical representation of these percentages.

### Identifying common aspects involved in data hub governance

Below, a general pattern of data governance for data hubs is presented, defining common aspects involved in the data hubs’ governance models, using the conclusions obtained in the analysis of the 41 survey responses and taking into account the list of more frequent aspects. Data aspects, business models and ELSI aspects are defined, preceded by the list of actors involved in these processes.

#### Actors

In a data hub, the data controller refers to the ‘party that, alone or jointly with others, determines the purposes and means of the processing of personal data’ [17]. Depending on the data hub, there may be one or more data controllers and sometimes there is a data controller for each dataset. The data controller can be any institution, such as a research institute, university, hospital, health service, etc.

The data processor determines who is in charge of data processing, ‘which processes personal data on behalf of the controller’ [17]. This actor can vary depending on the particular case: it can be the same data hub, another institution, or there can be no data processor.

Regarding the organization’s role in personal data, data hubs can be data controllers or data processors. They also can have both roles depending on the specific situation.

The data access provider is defined as ‘an entity which makes data available for secondary use’ [17]. There may be one or several; it may be a person or a set of mechanisms. Other relevant actors such as researchers, ethical and scientific committees, advisory committees, management boards (government bodies that evaluate applications) or data protection agencies, among others, can be found.

#### Data aspects

Concerning data characteristics that are frequently present in the data hubs, these kinds of data infrastructures usually: (i) are digital platforms that receive and store data, (ii) have control over the stored data, receiving data from a single source and/or multiple sources, (iii) are a digital technical infrastructure with the core mission of enabling health data sharing and providing health services data from different sources enabling the discovery of health datasets by having a published metadata discovery service and data accessibility mechanism in accordance with existing regulation that has an authorization functionality, and (iv) provided by the data hub itself or by an external institution. In addition, although less common, a data hub can have characteristics such as generating data, being part of one or more overarching data hubs, or having a specific thematic or collected data type (e.g. a particular disease, a particular data type, etc.), amongst others.

Related to the geographical coverage of the data infrastructure, it can be national, which is the most common, or European, regional or international, which is with less frequency.

As far as the organization of the data infrastructure, the most common is in a centralized way, and less frequently in a decentralized (federated) way. A data hub can also be part of another data hub, although this characteristic is not very frequent.

Regarding the origin of the data, health data usually come from the general population or from a patient group. With less frequency, health data come from an experimental setting, amongst others.

Common types of data sources are EHRs, administrative data, registry data and healthcare data, such as prescriptions, diagnoses, laboratory data, treatment, surgery, etc. Nevertheless, other types of data sources can also be clinical trials, surveys, cohorts, biobanks (biological samples), picture archiving and communication system (PACS), imaging data, medical devices, clinical research data, genomic data, biometric data, molecular data, socioeconomic data, specific disease data, survival data, population health data, interview data, customer record data or observational study data, amongst others less common.

Related to the level of aggregation of the data stored (individual versus aggregated), the data hubs frequently present an individual level or both, but it also (although less common) can be only aggregated. Concretely, the data hubs were asked about data access to individual and/or aggregated data by third-party users, and 24/41 provided access to both individual and aggregated data, 10/41 provided access to only individual data and 4/41 provided access to only aggregated data. Analysing the respondents’ answers, the conclusion related to the feasibility of linking individual-level data between data collections is that the recommendation is to provide access to individual data and aggregated data for third-party users. Aggregated data provides information, but the higher the level of granularity, the more possibilities for reuse, so it is also interesting to share individual data when possible.

Most of the data hubs have a funding sustainability plan. The data hub can receive national funding (most common), or international, regional, from a hospital, European, related to participation in projects or private funding.

Data hubs can receive data from different sources, providing a catalogue of these different data sources. Data are shared through a website, a secure data exchange portal, Application Programming Interface (APIs), File Transfer Protocol (FTP), Secure File Transfer Protocol (SFTP), Digital Imaging and Communications in Medicine (DICOM) transfer, amongst other options. This characteristic can depend on the specific usage request.

#### Business processes

Related to how the data are compiled and stored in the data hub, data retrieval; loading, extract, transform, load (ETL) methods; transforming; or passing, amongst others, can be used. The storage can be supported by technologies such as Structured Query Language (SQL), relational database, Sorl, MongoDB, Oracle, Cloud data lakes, DataOntap, DICOM, XML, Resource Description Framework (RDF), comma-separated values (CSV), JavaScript Object Notation (JSON), DBs, or a self-developed database/geographic information system. Data can be stored in several formats such as plain text, XML, or files (the most common), but also in others such as JSON, DICOM, tsv, RDF, FASTA, Dublin core, Parquet, Nifti, Fast Healthcare Interoperability Resources (FHIR), Oracle tables, OMOP Common Data Model, SAS Data Set, etc.

Data hubs usually apply quality controls to their data and require a minimum level of data quality to be included in the data infrastructure. Sometimes, a data hub applies quality controls only for internal use. Frequently, passing quality control is not mandatory for the data, but the results of quality control are available when searching the data. It is relevant for data hubs to use tools for checking errors and completeness of data. The most used is Checksum, but there are also many others such as HEX/SHACL, XSD Schemas, SQL-Scripts, R-dlookr, or even an automatic web-based check, a data submission portal and manual checks of certain variables or a specific software developed for the purpose of the network, or other options. Data hubs with low frequency use methods to check data source legitimacy, such as a Data Utility Framework, accreditation of the data provider institute, an authentication of the data providing individual, quality/FAIRness/sustainability assessments, etc.

Related to how often the datasets are updated, this characteristic depends on each specific dataset, and the most usual is to update annually, daily or irregularly, although they can also be updated monthly, weekly or even every 12 h, amongst others. Another option is to perform a one-time collection without updates.

Data hubs have processes to keep track of the different versions of datasets, such as manually creating versions by saving the date and name of each update, applying a different PID each time a version is stored, tracking model or software changes documented in the metadata management, or storing it in the log history. In addition, each data type may have a different process for versioning.

On the subject of describing the logging and auditing of user actions, data hubs can time stamp the data deposition, time stamp the user contact to client service, and/or time stamp the user application to download or see the health data.

Data hubs commonly have a formal procedure to know who provides the data, practically materialized in contracts, agreements, regulations, terms of use, licence, accreditation–authentication, alliance membership, a law framework making formal requests for data collection mandatory approvals, records on data processing and provision, amongst others. It is also important to highlight that data hubs frequently establish standard operating procedures (SOPs) that the organization follows and updates regularly.

It is highly recommended for data hubs to include a Data Governance section describing the data governance model used on their websites; it can be in the form of a detailed document or in a paragraph.

#### ELSI aspects

Concerning ethical aspects, before accepting new submissions, data hubs may require ethical approval for data to be stored in the infrastructure. After receiving ethical approval, the submission can be completed.

Related to anonymization and pseudonymization of data, data hubs usually use anonymization methods. The data can arrive already anonymized, which is not the most common. Additionally, the data hub itself can be in charge of anonymization. The process can be done at the point of collection, before sharing it externally (these two are the most common), before sharing it internally, or at the point of publication. Almost all data hubs pseudonymize their data; this can be done by the data hub itself or by another external organization.

Related to the legal aspects, when a data requester asks to access data and a data provider accepts the specific request, data hubs may offer a Data Access Agreement (DAA) to be signed between data providers and data requesters. It also can be done by data permission or by accepting a use policy. Data hubs may have a Data Processing Agreement (DPA) to be signed with the data providers, but it also can be by accepting use policy or depending on contracting situations. In addition, data hubs may have a Data Protection Impact Assessment (DPIA) model.

Data hubs usually implement mechanisms to control the access of the data (authentication and authorization) such as authorization with web services backed by a database, OAuth2, OpenID Connect (over HTTPs), or other options.

### Profiling kinds of data hub organization

To cover this profiling, the question ‘How is the data infrastructure organized?’ was analysed. From the 41 surveyed data hubs, 40 answered and 1 did not answer. Of these 40 responses, 30 (75%) answered ‘it is managed centrally’, 9 (22%) answered ‘it has a decentralized management’, and 1 (2%) ‘this does not apply to this data infrastructure’.

Using the conclusions obtained in the stratifications in terms of the kind of organization, two profiles were described: ‘data hubs managed centrally’ and ‘data hubs have decentralized management’. The specifications or peculiarities of these profiles compared with those described in the general pattern of data governance are presented in Table 1.

**Table 1:** Profiles depending on the kind of data hub organization

	Data hubs managed centrally	Data hubs having decentralized management
Actors	No peculiarities	May not a single data controller No data management strategy
Data aspects	Control the data stored Data from ‘general population’ Use ‘text’, ‘numbers’ Receive and store data from: single source, multiple sources	Data from ‘patient groups’, ‘general population’, ‘experimental settings’ Use ‘text’, ‘images’, ‘numbers’ Data stored in ‘XML’
Business processes	Data quality control SOPs Procedure to know who provides data​​	No peculiarities
ELSI aspects	Pseudonymized data Require legal approval	No peculiarities

### Profiling roles

For this profiling, the question ‘What is your organization’s role in relation to personal data?’ was analysed. From the 41 surveyed data hubs, 39 answered and 2 did not answer. Of these 39 answers, 11 (28%) answered ‘data controller’, 11 (28%) answered ‘data processor’, 12 (33%) answered ‘we have different roles in different situations’ and 4 (10%) answered ‘none of the above’.

Using the conclusions obtained in the stratifications in terms of the organization role, two profiles were described: ‘data hubs acting as data controller’ and ‘data hubs acting as data processor’. The specifications or peculiarities found of these profiles compared with those described in the general pattern of data governance are presented in Table 2.

**Table 2:** Profiles depending on the role performed by the data hub

	Data hubs acting as data controller	Data hubs acting as data processor
Actors	No peculiarities	No peculiarities
Data aspects	Managed centrally Pseudonymized data Receive and store data from: single source, multiple sources Data from ‘general population’ Use ‘text’	Managed centrally Receives and stores the data Functional authorization Data accessibility mechanism in accordance with existing regulations
Business processes	Procedure to keep track of datasets versions. Procedure to know who provides data​​	Procedure to know who provides data​​
ELSI aspects	SOPs Catalogue of data sources	SOPs Pseudonymized data

## Discussion

Recent advances in big data are expected to expand our knowledge to test new hypotheses about disease management, from diagnosis to prevention to personalized treatment. However, the rise of big data also poses challenges in terms of privacy, security, data ownership, data stewardship and governance [20]. In addition, the wide availability of data has led to the need for additional attention to the health research field, where the number of studies seeking to leverage data to improve healthcare has grown significantly. Healthcare data are increasingly complex and are obtained in a variety of ways, from a variety of sources, contexts and technologies, and their nature can impede proper analysis. Any analytical research must overcome these obstacles to extract data and produce meaningful insights. Hence the importance of investigating the main challenges, data sources, techniques and technologies, as well as future directions in the field of big data analytics in healthcare [21].

The basis of the study performed has been that accommodating data hubs with different governance models is essential to enabling the decentralized ecosystem for health research across Europe. Health data reuse is widely used in healthcare, research, government and business settings. Studying the benefits, barriers to use with large clinical databases, policy frameworks that have been formulated, and challenges makes the study of data management and governance essential to promote data sharing and reuse [22, 23]. To address the purpose of this study, health data management [24] and health data hub patterns of data governance were defined through the analysis of a dedicated survey of a representative list of national, European, and global health data hubs.

On the one hand, a general pattern of data governance for data hubs was defined using the findings obtained in the analysis of the 41 survey responses, detailing the most frequent aspects of health data hubs analysed and identifying actors and business processes involved in data hub governance. On the other hand, specific data governance patterns were generated through the stratifications in terms of the kind of organization (centralized versus decentralized) and role (controller or processor). Specific profiles were defined including the actors involved, data and ELSI aspects, and business processes. In this regard, it is important to clarify that the recommendations from the survey analysis were included with the term anonymization, and not de-identification as is recommended in other fields [25], as the survey question was aimed at finding out whether all personal identifiable information in data hubs is removed.

In addition, this is the first study that presents relevant best practices on data management and governance, taking into account the information provided by health data hubs through the evaluation of the survey responses.

Particular attention was paid to understanding the potential limitations and constraints of existing governance models that resulted in a number of breakthroughs in the medical field [26–28]. Most of the data hubs include related costs to access the data as part of their data governance model. This limitation slows down the progress in open science [29–31]. The time spent for ethical approval and for accessing the data themselves is a constraint in the final use of the data. In some cases, the absence of a sustainability plan was identified. This fact endangers the continuity of the data infrastructures. To ensure working in a secure environment, anonymization and/or pseudonymization methods, and logging and auditing mechanisms including access control mechanisms (authentication and authorization) must be used. Finally, it is relevant to mention that, to have high-quality data, tools, processes or methods must be applied in terms of error checking, completeness, version tracking and legitimacy. Not all data hubs cover these kinds of mechanisms.

In terms of limitations in the study execution, it is relevant to mention the difficulties to identifying the list of representative data hubs due to the nonexistence of a repository of contacts for the representative data hubs in Europe. Additionally, the participation of the data hubs through a survey was not easy due to availability matters (41% of the contacted data hubs answered the survey). In terms of analysing the responses, in the case of non-mandatory questions, some data hubs did not complete some questions, 35 questions offered the possibility to include free text (directly answering the question, or through the ‘other’ option in a structured question), adding a subjective interpretation in the analysis, 4 of these 35 free-text questions asked for URLs linking to a lot of material to explore, and some free-text responses could not be used due to problems in interpretation (e.g. an estimation of size specifying the number without specifying the unit).

Finally, to strengthen the results and to promote participation, the list of best practices was validated with the data hubs interviewed, involving them in the review phase of the best practices generated. In that stage, another important conclusion was identified: smaller decentralized data hubs may struggle to implement all the recommendations in a short timeframe.

## Conclusions

The findings gathered in the survey analysis of the survey responses, as well as the list of the most frequent aspects presented in the results section, facilitated a list of best practices proposed for health data hubs. Specific profiles generating specific data governance patterns for health data hubs were defined in this study. Furthermore, it is relevant to highlight that the governance models discovered in this study were validated with the health data hubs respondents, involving them in the review phase of the governance patterns.

The most relevant best practices on data management and governance that must be considered together with the constraints of sensitive data were identified by analysing the list of the most frequent aspects of data hubs respondents.

After analysing the results of the survey, and on the basis of the landscape analysis it provides, together with literature reviewed as background in this manuscript, a set of good practices for data governance in health data hubs is concluded and drafted in Table 3.

**Table 3:** The most relevant best practices on data governance for health data hubs

Best practices	Description/example
Configure your data hub in a centralized way	That is, it requires a connection process for whom the data hub receives and stores the data directly. For example, a specific data hub has the control of the data stored and can receive and store data from a single source and/or from multiple sources
Complete and sign a Data Processing Agreement (DPA)	The DPA includes the data use policy and contracting situations, as well as the agreed terms between the data access provider and data processor in terms of processing
Apply mechanisms of quality control to the data	For instance, a data hub can include data only if it reaches a certain quality level or performs data quality controls for internal use
Define a formal procedure to find out who provides the data	In this sense, for data management it is relevant to know who provides the data through a formal procedure (i.e. legal contracts, agreements, or open information in the organization)
Provide a catalogue of the different data sources	For example, that catalogue is really useful in the case of a data hub that connects to several data sources
Apply anonymization and/or pseudonymized methods	For instance, in the case of health data hubs that do not receive anonymized data, anonymization and/or pseudonymized methods are recommended as applicable to comply with general data protection regulation (GDPR) rules [32]
Use any tool to check for errors and data integrity	This best practice is included because checking for errors and completeness is another important aspect of data quality in data hubs. For example, tools such as Checksum, HEX/SHACL, XSD Schemas, SQL-Scripts, R-dlookr, or even an automatic web-based check, a data submission portal and manual checks of certain variables or a specific software developed for the purpose of the network
Include in the data hub website a data governance section describing the data governance model used	Important information related to the data governance model or data management can be provided by data hubs through their websites

## Supplementary Information

**Additional file 1:** Survey questions and list of respondents.

## Data Availability

Not applicable.

## Abbreviations

DAA: Data access agreement
DPA: Data processing agreement
DPIA: Data protection impact assessment
EHDS: European health data space
EHR: Electronic health records
ELSI: Ethical, legal, societal impact
GDPR: General data protection regulation
HealthyCloud: Health research & innovation cloud
HRIC: Health research and innovation cloud
SOPs: Standard operating procedures
