# Supplementary material for: Health data hubs: an analysis of existing data governance features for research
Source: Health Res Policy Syst. 2023 Jul 10;21:70. doi: 10.1186/s12961-023-01026-1 (PMC10332005; doi:10.1186/s12961-023-01026-1)
Supplement: Supplementary file 1 — Additional file 1: Survey questions and list of respondents. [file 12961_2023_1026_MOESM1_ESM.docx]

Additional file 1

Below, the survey questions that were sent to health data hubs identified in the study are included.


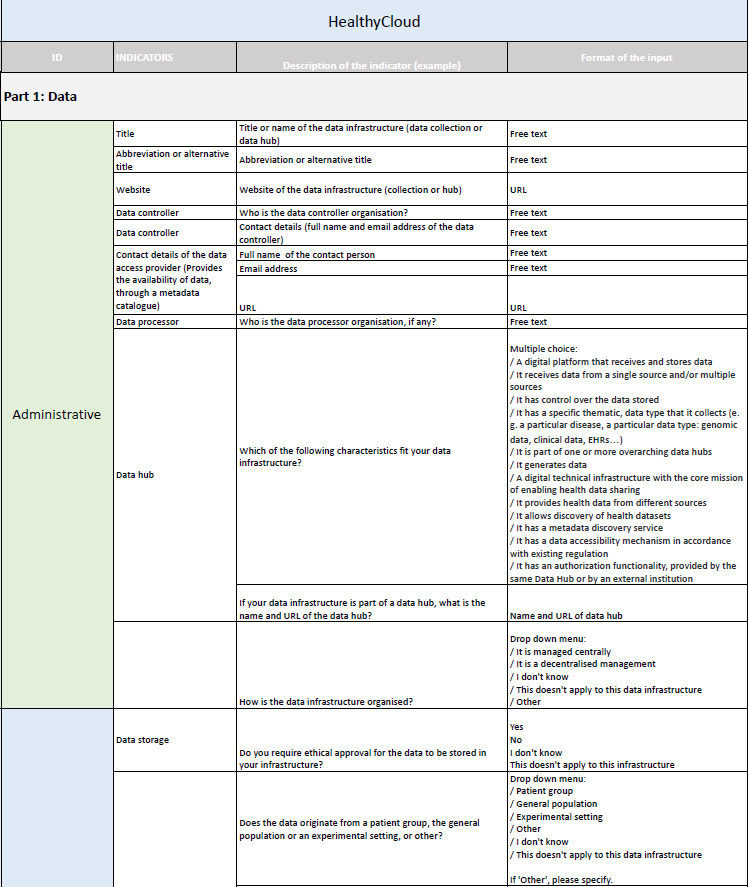


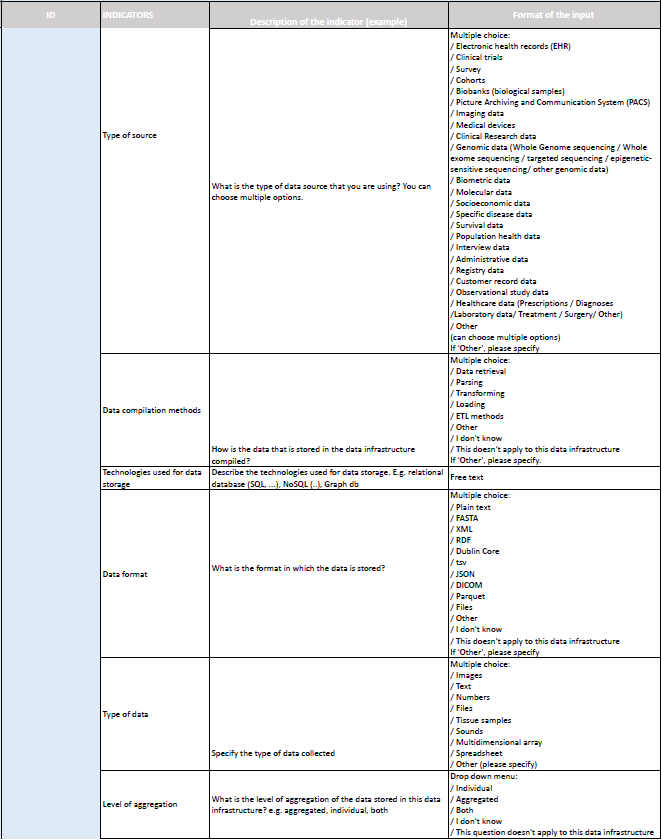


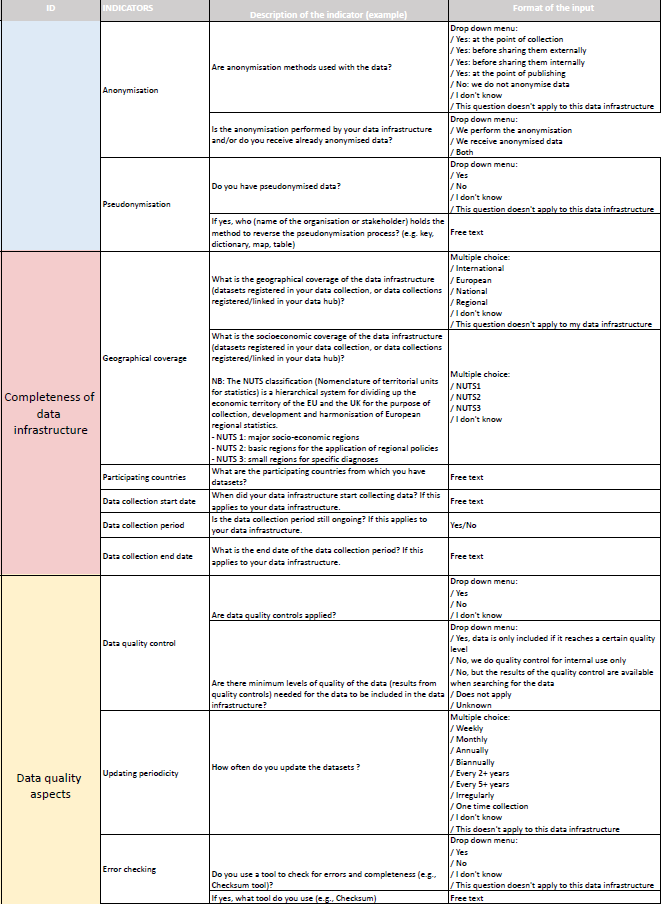


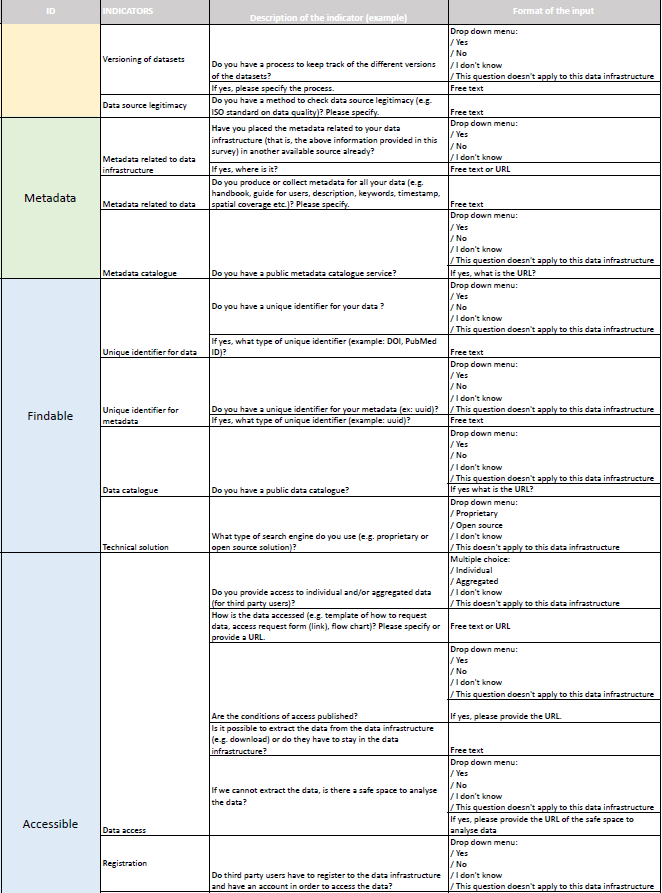


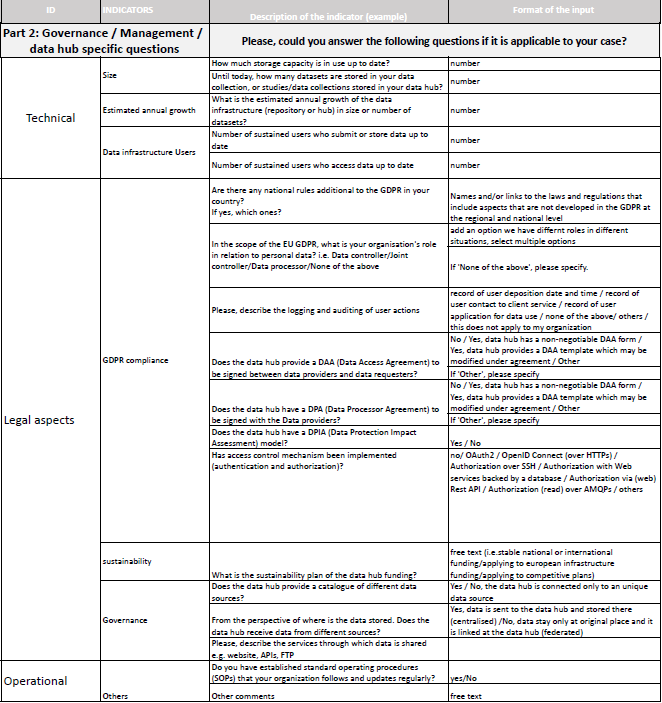


In addition, a detailed list of the 41 respondents data hubs is shown.

| Name | Abbreviation | Website | Country |
| --- | --- | --- | --- |
| European Joint Programme on Rare Diseases Virtual Platform for discoverable Data & Resources for RD research | EJP RD VP | [https://vp.ejprarediseases.org](https://vp.ejprarediseases.org/) | European |
| Medical Data Integration Center of the University Hospital rechts der Isar of the Technical University of Munich | MeDIC MRI/TUM | <https://www.mediz.med.tum.de/> | Germany |
| Health Data Lab (Forschungsdatenzentrum Gesundheit) | HDL (FDZ) | <https://www.forschungsdatenzentrum-gesundheit.de/> | Germany |
| Plataforma de Información BIGAN | BIGAN | [https://bigan.iacs.es](https://bigan.iacs.es/) | Spain |
| EUROCAT Central Registry | EUROCAT | <https://eu-rd-platform.jrc.ec.europa.eu/eurocat_en> | European |
| SCPE Central Registry | SCPE | <https://eu-rd-platform.jrc.ec.europa.eu/scpe_en> | European |
| European Rare Disease Registry Infrastructure (ERDRI) - (part of the European Platform of Rare Disease Registration) | ERDRI - EU RD Platform | <https://eu-rd-platform.jrc.ec.europa.eu/erdri-description_en> | European |
| Clinical Practice Research Datalink | CPRD | [https://www.cprd.com](https://www.cprd.com/) | Worldwide |
| Data Integration Center Dresden | DIZ | <https://www.uniklinikum-dresden.de/de/das-klinikum/universitaetscentren/zentrum-fuer-medizinische-informatik/zentrum/organisation-des-zentrums/datenintegrationszentrum-diz> | Germany |
| National Infectious Disease Register | NIDR | <https://thl.fi/en/web/infectious-diseases-and-vaccinations/surveillance-and-registers/finnish-national-infectious-diseases-register> | Finland |
| Research Services at Statistics Finland | NA | <https://www2.tilastokeskus.fi/tup/mikroaineistot/index_en.html> | Finland |
| Data Platform COVID-19 | NA | <https://datenplattform-covid.goeg.at/> | NA |
| BBMRI-ERIC | NA | <https://www.bbmri-eric.eu/> | Worldwide |
| Datenintegrationszentrum | DIZ | <https://www.ukw.de/zentrale-einrichtungen/servicezentrum-medizin-informatik-smi/datenintegrationszentrum-wuerzburg-diz/> | Germany |
| Italian Multi-sited Multi-Modal Molecular Imaging (MMMI) Node | MMMI | <http://www.mmmi.unito.it/it/content/image-datasets> | Italy |
| BCU Imaging Biobank | BCU-IB | <https://bcuib.biocheckup.net/> | Italy |
| Austrian Integrated Health Information System | ÖGIS | <https://goeg.at/OEGIS> | Austria |
| IRCCS SYNLAB SDN - Biobank | SDN-BB | <https://sdn.synlab.it/facilities/> | NA |
| EUDAT CDI | EUDAT | [https://www.eudat.eu](https://www.eudat.eu/) | European |
| BioMedIT platform | BioMedIT | <https://sphn.ch/projects/biomedit> | Switzerland |
| EATRIS-ERIC | EATRIS | <https://eatris.eu/> | European |
| Finnish Social Science Data Archive | FSD | <https://www.fsd.tuni.fi/en/> | Worldwide |
| TMf e.V., Berlin acting on behalf of Medical Informatics Initiative Germany | MII | [https://www.medizininformatikinitiative.de](https://www.medizininformatikinitiative.de/) | Germany |
| Health-RI XNAT.bmia.nl | XNAT.bmia.nl | <https://www.health-ri.nl/services/xnat> | Netherlands |
| Avohilmo, Register of Primary Health Care Visits | Avohilmo | <https://thl.fi/fi/tilastot-ja-data/ohjeet-tietojen-toimittamiseen/perusterveydenhuollon-avohoidon-hoitoilmoitus-avohilmo> | Finland |
| Health Data Research UK | HDR UK | [https://hdruk.ac.uk](https://hdruk.ac.uk/) | Worldwide |
| DisGeNET | Knowledge discovery platform on disease genomics | <https://www.disgenet.org/> | NA |
| THL Biobank | No abbreviation | <https://thl.fi/en/web/thl-biobank> | Finland |
| Base de Datos para la Investigación Fármaco-epidemiológica en Atención Primaria | Programa BIFAP | <http://bifap.aemps.es/> | Spain |
| Findata | Social and Health Data Permit Authority Findata | <https://findata.fi/en/> | Finland |
| PIONEER | NA | NA | Worldwide |
| Elixir Translational Medicine Data hub | Elixir Transmed Data Hub | <https://elixir-europe.org/about-us/who-we-are/nodes/luxembourg> | Worldwide |
| European Genome-phenome Archive | EGA | <https://ega-archive.org/> | Worldwide |
| Causes of death statistics | CoD | <http://www.statistik.at/web_de/statistiken/menschen_und_gesellschaft/gesundheit/todesursachen/index.html> | Austria |
| The Danish Clinical Quality Program– National Clinical Registries (RKKP) | RKKP | <https://www.rkkp.dk/in-english/> | Denmark |
| National Public Health Information System | NAJS | <https://www.hzjz.hr/nacionalni-javnozdravstveni-informacijski-sustav-najs/> | Croatia |
| Estonian Biobank | EstBB | [https://www.genomics.ut.ee](https://www.genomics.ut.ee/) | Estonia |
| France Cohortes | NA | <https://francecohortes.org/> | France |
| Polish Platform of Medical Research - Researcher Data | PPM | <https://ppm.edu.pl/globalResultList.seam?r=researchdata&tab=RESEARCHDATA&lang=en> | Poland |
| EESZT (Hungarian eHealth Service Space) | EESZT | [https://www.eeszt.gov.hu](https://www.eeszt.gov.hu/) | Hungary |
| Central IT Platform for Polish Biobanking Network | BBMRI.pl Central Platform | [https://biobanks.pl](https://biobanks.pl/) | Poland |
